# Feasibility study of in‐house second‐channel calibration of dual‐channel electrometers using a battery‐powered current source

**DOI:** 10.1002/acm2.70476

**Published:** 2026-03-05

**Authors:** Hayato Tsuno, Ruan Sasaki, Koji Sasaki, Kohei Nishi, Tae Ushikawa, Daisaku Goto, Yasuhiro Kawashima, Ririko Kosugi

**Affiliations:** ^1^ Gunma Prefectural College of Health Sciences Gunma Japan; ^2^ Japanese Red Cross Maebashi Hospital Gunma Japan; ^3^ Graduate School of Radiological Technology Gunma Prefectural College of Health Sciences Gunma Japan; ^4^ JR Tokyo General Hospital Tokyo Japan

**Keywords:** current source, dosimeter, dual‐circuit electrometer, electrometer, second‐circuit calibration

## Abstract

**Background:**

An ionization chamber and electrometer allow measurement of the absorbed dose to water. A sensitivity comparison between electrometers is essential for quality control, and an efficient method is available to accurately measure the electrometer sensitivity coefficient without using a linear accelerator (linac). Although dual‐ circuit electrometers are becoming increasingly common, no calculation method for the sensitivity coefficient of their second‐circuit is available. Hence, we propose a method for calculating this sensitivity coefficient using the first‐circuit as the reference and evaluate its accuracy.

**Methods:**

Using the first‐circuit of a RAMTEC pro electrometer as a reference, the RAMTEC duo and SuperMAX electrometers were connected as test units to the simple yet accurate Japanese‐patented SCG002 current source powered by a dry cell battery. Sensitivity ratio *r*_elec_​ was calculated from the average of three charge measurements using RAMTEC Pro. This ratio was multiplied by the calibration coefficient of the first‐ circuit to obtain the sensitivity coefficient of the second‐circuit. The accuracy was obtained from the relative error of each electrometer based on the calibration coefficient (*k*_elec_) provided by a standards laboratory.

**Results:**

The sensitivity coefficient of the second‐circuit of RAMTEC pro was 1.0004 (relative error, +0.030%). For RAMTEC duo, the first‐ and second‐circuit coefficients were 1.0014 and 1.0013, respectively (relative errors, +0.080% and +0.070%). For SuperMAX, the coefficients were 0.9986 and 0.9983 (relative errors, 0.0% and –0.050%) for the first and second circuits, respectively. Thus, the proposed method provided accurate measurements.

**Conclusion:**

We accurately determine the sensitivity coefficient of the second‐circuit in a dual‐circuit electrometer using the first‐circuit of the same or another electrometer as the reference. If the electrometer performance is verified, the coefficient *k*_elec_ of the first‐circuit is likely applicable to the second‐circuit. This method may reduce the costs associated with electrometer calibration in clinical settings.

## 1 INTRODUCTION

In external beam radiation therapy, the measurement of absorbed dose‐to‐water is essential, and the employed ionization chambers and electrometers must be calibrated by a standards laboratory.^1^, ^2^, ^3^ These systems are calibrated separately under defined conditions, and electrometers require calibration every few years.^4^ For electrometer response checks, radiation therapy facilities generally need to use the beam output from their own linear accelerator (linac). To minimize variability in beam output, an external monitoring dosimeter should be installed.^2^ This approach resembles the cross‐calibration of ionization chambers, requiring two sets of chambers and electrometers.^5^ Consequently, facilities equipped with only one set of ionization chamber and electrometer face difficulties in performing such checks.^5^

To enable facilities with only one set of equipment to easily verify electrometer response ratios, our research team developed a current source using a dry cell battery and obtained a Japanese patent for it.^6^ The corresponding method does not require a linac and thus does not need an ionization chamber. Additionally, it uses a simple direct current from a battery without relying on an alternating‐current power source.

In our method, the electrometer calibration coefficient (*k*_elec_) previously determined by a standards laboratory serves as the reference to determine the relative sensitivity (*r*_elec_) of another electrometer. By multiplying *r*_elec_ by *k*_elec_, the electrometer sensitivity coefficient (*k*_elec,user_) of the second electrometer can be determined with an accuracy equivalent to that of official calibration, thus being suitable for clinical use.^5^, ^6^, ^7^

In this paper, we define *k*_elec_ as the calibration coefficient assigned by a standards laboratory and *k*_elec,user_ as the coefficient determined from the sensitivity ratio. Coefficient *k*_elec_ may vary by 0.25%,^8^ and the expanded uncertainty (*k* = 2) of *k*_elec,user_ is 0.36%, with both values subject to ongoing debate.^7^ Moreover, because our method does not require a linac, electrometer quality assurance can be performed without disrupting patient treatments, thereby improving the workflow efficiency.^7^

Recently, electrometers equipped with dual measurement circuits in a single unit have become widespread. However, the feasibility of using our current source has not been evaluated to compare the calibration coefficients of the second circuit r with those of the first circuit. As we consider this a pressing issue, this study was aimed to demonstrate that the calibration coefficient of the second circuit in a dual‐circuit electrometer can be determined by intercomparing circuit responses.

## 2 METHODS

### 2.1 Materials

The SCG002 current source (Kawaguchi Electric Works, Tokyo, Japan) was used in this study. This device was granted a Japanese patent.^6^ Due to the high linearity between the battery voltage and current output (coefficient of determination *R* ^2^ of 1.00),^5^ fluctuations in current caused by battery degradation can be neglected.

To evaluate the temperature response, a thermostatic chamber was set at 15°C, 18°C, 23°C, and 35°C. After placing the current source inside the chamber for 24 h, the maximum absolute change in current was 5.968 × 10^−^^5^ nA (i.e., 5.968 × 10^−^^5^ nC/s), and the relative change per 1°C remained below 0.013%.^5^ Additionally, the maximum relative change after 60 min was −0.056%.^5^ These results confirm that SCG002 has high linearity, minimal temperature dependence, and negligible temporal drift. The polarity of the current output from SCG002 can be changed to positive or negative. Because the SCG002 is equipped with two resistors, two current modes can be set. These will be referred to as high mode and low mode, respectively.

The electrometers used in this study were RAMTEC Pro (Toyo Medic, Tokyo, Japan), RAMTEC Duo (Toyo Medic), and SuperMAX Electrometer (Standard Imaging, Middleton, WI, USA). All electrometers had dual measurement circuits labeled first‐circuit and second‐circuit, and in the formulas below, labeled #1 and #2, respectively.

For the RAMTEC Pro and RAMTEC Duo, only the first‐circuit is calibrated by a standards laboratory. For the SuperMAX, both the first and second‐circuits are calibrated by a standards laboratory. Table 1 lists the *k*_elec_ used in the study. In accordance with the aforementioned rules, the symbols for the first‐circuit *k*_elec_ are $k_{\mathrm{elec}}^{\#1}$ and the second‐circuit *k*_elec_ are $k_{\mathrm{elec}}^{\#2}$.

**TABLE 1:** The electrometer calibration coefficients for the electrometers used in this study were calibrated by a standard laboratory. $k_{\mathrm{elec}}^{\#1}$ and $k_{\mathrm{elec}}^{\#2}$ are the electrometer calibration constants for the first‐ and second‐circuits, respectively.

Electrometer	RAMTEC Pro	RAMTEC Duo	SuperMAX
$k_{\mathrm{elec}}^{\#1}$	1.0001	1.0006	0.9986
$k_{\mathrm{elec}}^{\#2}$	–	–	0.9988

**FIGURE 1:**
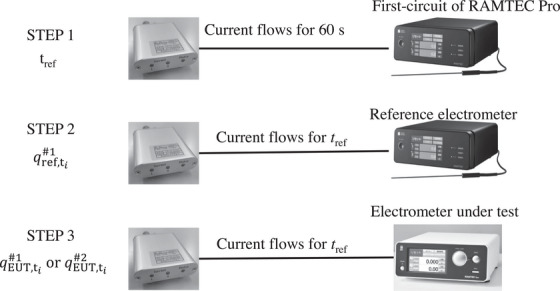
Conceptual diagram of this research model. Step 1: Pass a current through the reference electrometer for 60 s and calculate the current value and *t*_ref_. *t*_ref_ is the calibration point in a standard laboratory; details are shown in Table 2. Step 2: Pass a current through *t*_ref_ to the reference electrometer and measure its sensitivity. Step 3: Pass a current through *t*_ref_ to the EUT and measure its sensitivity. Calculate the sensitivity ratio from the two measurement results.

An overview of this research is shown in Figure 1. First, in preparation for a mutual comparison of the sensitivities between electrometers, SCG002 was connected to a reference electrometer, and a current was applied for 60 s. From this measurement result, the current application time *t*_ref_ was calculated. The method for calculating *t*_ref_ is explained in 2.2 below, and only an overview will be given here. Second, a current was applied to the reference electrometer for the calculated current application time *t*_ref_, and the sensitivity of the reference electrometer was measured. Third, a current was applied to the electrometer under test (EUT) for the current application time *t*_ref_, and the sensitivity of the EUT was measured. Electrometer calibration coefficient *k*_elec_ was used as a reference, and EUT was evaluated by intercomparison. The sensitivity ratio was determined and multiplied by *k*_elec_ to obtain the electrometer sensitivity coefficient, *k*_elec,user_.

**FIGURE 2:**
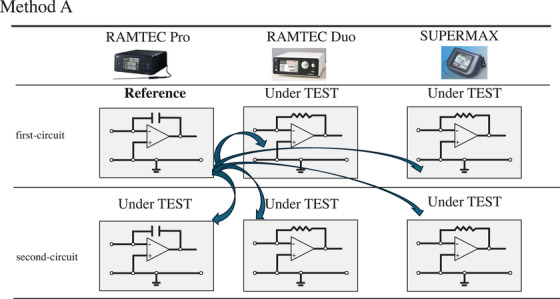
Conceptual diagram of method A. The sensitivity of first‐circuit of the RAMTEC Pro electrometer is used as the reference. Electrometer sensitivity coefficient *k*_elec,user_ is measured using second‐circuit of RAMTEC Pro and first‐circuit and second‐circuit of both RAMTEC Duo and SuperMAX as EUTs.

**FIGURE 3:**
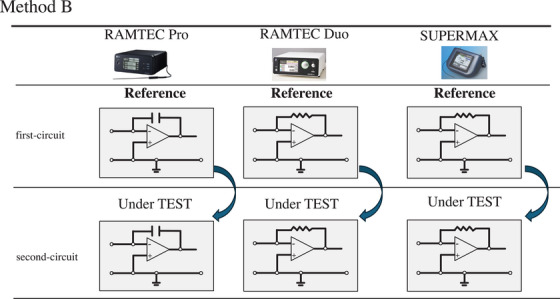
Conceptual diagram of method B. The sensitivity of first‐circuit of every electrometer is used as the reference to determine sensitivity coefficient *k*_elec,user_ for second‐circuit. This method has a radiotherapy facility that possesses only one dual‐circuit electrometer and uses first‐circuit of the same unit as the internal reference.

Two methods were used to define the reference electrometer in this study. In method A (Figure 2), first‐circuit in RAMTEC Pro was used as the reference, while second‐circuit in RAMTEC Pro and first‐circuits and second‐circuits in both RAMTEC Duo and SuperMAX were the EUTs. In method B (Figure 3), first‐circuits in RAMTEC Pro, RAMTEC Duo, and SuperMAX were used as references, while second‐circuits in RAMTEC Pro, RAMTEC Duo, and SuperMAX were the EUTs. The intercomparison procedures are detailed in Section 2.2.

### 2.2 Methods

#### 2.2.1 Current measurement procedure

The SCG002 current source was connected to first‐circuit in RAMTEC Pro, and current was applied over five trials^7^ for 60 s per trial. At this time, the SCG002 was set to high and low current modes, and the polarity modes were combined to positive and negative. The coefficient of variation *CV* was calculated using Equation (1), and the current *I* of the SCG002 source was calculated using Equation (2).

(1) $$\begin{array}{c} \mathrm{CV}=\frac{\mathrm{SD}}{{\bar{q}}_{\mathrm{ref},60s}^{\#1}}\;\times 100\;\left[\%\right] \end{array}\;$$

(2) $$\begin{array}{c} I=\frac{{\bar{q}}_{\mathrm{ref},60s}^{\#1}}{60s}\;\left[\mathrm{nC}/s\right] \end{array}$$

where ${\bar{q}}_{\mathrm{ref},60s}^{\#1}$ is the mean electric charge measured by first‐circuit in RAMTEC Pro (reference electrometer) over 60 s and *SD* is its standard deviation. Next, using current *I* and electric charge *Q_i_*, the required current application time, *t_i_*, was calculated as follows:

(3) $$t_{i}=\frac{Q_{i}}{I}\left[s\right]\left(i=\min,\;\max,\;0.5\right)$$

The standards laboratory performed electrometer calibration at three charge points: *Q*_min_, *Q*_max_, and *Q*_0.5_ (i.e., half of *Q*_max_). *Q*_min_, *Q*_0.5_, and *Q*_max_ used when calibrating by the standards laboratory are shown in Table 2. For all electrometers used in this study, they were 1 nC, 100 nC, and 200 nC, respectively. Current *I* obtained after application for *t_i_* seconds to the reference electrometer was denoted as $q_{\mathrm{ref},t_{i}}^{\#1}$​. Second‐circuit was not used as the reference in either method A or B, with first‐circuit being consistently used as the reference.

**TABLE 2:** Calibration charge amounts are calibrated by a standard laboratory for the electrometer used in this study.

Electrometer	RAMTEC Pro	RAMTEC Duo	SuperMAX
*Q*_min_ [nC]	1	1	1
*Q*_0.5_ [nC]	100	100	100
*Q*_max_ [nC]	200	200	200

Similarly, the electric charges measured after applying the same *t_i_* current to the EUT were defined as $q_{\mathrm{EUT},t_{i}}^{\#1}$ and $q_{\mathrm{EUT},t_{i}}^{\#2}$ for first‐circuit and second‐circuit, respectively.

#### 2.2.2 Calculation of coefficient *k*_elec,user_

The sensitivity ratio of the EUT was defined as the ratio of the reference electrometer charge to the EUT charge at time *t_i_* ​ and calculated as follows:

(4) $$r_{\text{elec},t_{i}}^{\#1}=\frac{q_{\text{ref},t_{i}}^{\#1}}{q_{\text{EUT},t_{i}}^{\#1}}\left(i=\min,\;\max,\;0.5\right)$$

(5) $$r_{\text{elec},t_{i}}^{\#2}=\frac{q_{\text{ref},t_{i}}^{\#1}}{q_{\text{EUT},t_{i}}^{\#2}}\left(i=\min,\;\max,\;0.5\right)$$

In method A (Figure 1), $q_{\mathrm{ref},t_{i}}^{\#1}$ was measured using first‐circuit in RAMTEC Pro, while in method B (Figure 2), it was measured using first‐circuit in every electrometer. Calibration coefficient *k*_elec,user_ for the EUT was calculated as follows:

(6) $$\begin{array}{c} \;k_{\mathrm{elec},\mathrm{user}}^{\#1}={\bar{r}}_{\mathrm{elec}}^{\#1}\;\times \;k_{\mathrm{elec}}^{\mathrm{ref},\;\#1} \end{array}\;$$

(7) $$\begin{array}{c} \;k_{\mathrm{elec},\mathrm{user}}^{\#2}={\bar{r}}_{\mathrm{elec}}^{\#2}\;\times \;k_{\mathrm{elec}}^{\mathrm{ref},\#1} \end{array}\;$$

where ${\bar{r}}_{\mathrm{elec}}^{\#1}$ and ${\bar{r}}_{\mathrm{elec}}^{\#2}$ are the mean sensitivity ratios at the three charge points (*t*_min_, *t*_max_, and *t*_0.5_) for first‐circuit and second‐circuit, respectively.

**FIGURE 4:**
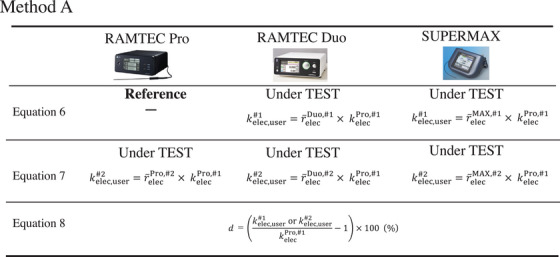
Detailed formula for calculating *k*_elec,user_ using Method A. Method A uses $k_{\mathrm{elec}}^{\mathrm{Pro},\#1}$ because it is based on the first circuit of the RAMTEC Pro.

**FIGURE 5:**
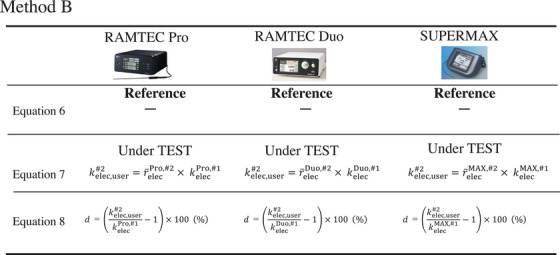
Detailed formulas for calculating *k*_elec,user_ using method B. Method B uses a different *k*_elec_ because it is based on the first circuit of each EUT.

The upper and middle rows of Figures 4 and 5 show the differences between equations (6) and (7) used in methods A and B. Method A uses first‐circuit of the RAMTEC Pro as the reference, so $k_{\mathrm{elec}}^{\mathrm{ref},\;\#1}$ in equations (6) and (7) is the $k_{\mathrm{elec}}^{\mathrm{Pro},\;\#1}$ of the RAMTEC Pro. On the other hand, the EUT in Method B is the respective electrometers, so $k_{\mathrm{elec}}^{\mathrm{ref},\;\#1}$ is the $k_{\mathrm{elec}}^{\mathrm{Pro},\;\#1}$, $k_{\mathrm{elec}}^{\mathrm{Duo},\;\#1}$, and $k_{\mathrm{elec}}^{\mathrm{MAX},\;\#1}$ of the EUT.

### 2.3 Accuracy evaluation of coefficient *k*_elec,user_

To evaluate the accuracy of the calculated calibration coefficient, *k*_elec,user_, relative error *d* was calculated using Equation (8) by comparing *k*_elec,user_ to the known electrometer calibration coefficient, *k*_elec_​. The result was then compared with the uncertainty stated in the calibration certificate:

(8) $$d\;=\left(\frac{k_{\text{elec},\text{user}}}{k_{\text{elec}}^{\text{ref},\#1}}-1\right)\times 100\left[\%\right]$$

where *k*_elec,user_ ​corresponds to either $k_{\mathrm{elec},\mathrm{user}}^{\#1}$ or $k_{\mathrm{elec},\mathrm{user}}^{\#2}$​ depending on the calculated value. The denominator is $k_{\mathrm{elec}}^{\mathrm{ref},\#1}$. SuperMAX is calibrated to $k_{\mathrm{elec}}^{\mathrm{ref},\#2}$ (Table 1), but we changed the denominator to $k_{\mathrm{elec}}^{\mathrm{ref},\#1}$ to match the other electrometers. (See the bottom of Figures 4 and 5)

### 2.4 Evaluation of short‐term reproducibility and uncertainty

To confirm the reproducibility of this study, we measured the $k_{\mathrm{elec},\mathrm{user}}^{\#2}$ of RAMTEC Pro and SuperMAX at three‐month intervals. The uncertainties of these *k*_elec,user_ were calculated and listed in a budget sheet.

## 3 RESULTS

Table 3 lists the results obtained by connecting the SCG002 current source to first‐circuit in RAMTEC Pro and applying current over five trials. The measured mean and standard deviation was 93.568 ± 0.0083 nC, and the coefficient of variation was 0.00089%, being well below the acceptable 0.1% threshold. Current *I* was 1.559 nC/s.

**TABLE 3:** Results of five repeated measurements using SCG002 current source connected to RAMTEC Pro. The current was applied for 60 s, and the variation in charge measurements as well as current *I* were evaluated. The SCG002 has two current modes and two polarity modes, and these were combined to measure the charge quantity.

Parameters	First‐circuit of RAMTEC Pro			
Current polarity	Positive		Negative	
Current mode	Low	High	Low	High
Power‐on time [sec]	60	60	60	60
${\bar{q}}_{\mathrm{ref},60s}^{\#1}$	9.376	93.568	−9.372	−93.588
SD [nC]	0.00091	0.00083	0.00042	0.00052
CV [%]	0.0097	0.00089	−0.0045	−0.00056
*I* [nC/s]	0.1563	1.559	−0.1562	−1.560

SD: Standard deviation.

CV: Coefficient of variation.

Table 4 lists the parameters obtained using method A, with first‐circuit in RAMTEC Pro serving as the reference electrometer. The calculated values of *t*_min_, *t*_0.5_, and *t*_max_ using Equation (3) were 6, 64, and 128 s, respectively. The charges measured at these times by the reference electrometer, $q_{\mathrm{ref},t_{\min}}^{\#1}$, $q_{\mathrm{ref},t_{0.5}}^{\#1}$, and $q_{\mathrm{ref},t_{\max}}^{\#1}$, were 0.94305, 99.85719, and 199.6918 nC, respectively. The corresponding values measured by EUT on first‐circuit were 0.9418, 99.689, and 199.486 nC for RAMTEC Duo and 0.945, 100.0, and 199.9 nC for SuperMAX, respectively. On second‐circuit, RAMTEC Pro recorded 0.94035, 99.80363, and 199.6011 nC, while RAMTEC Duo recorded 0.9418, 99.740, and 199.455 nC, and SuperMAX recorded 0.947, 99.94, and 199.8 nC.

**TABLE 4:** Parameters obtained from the intercomparison of electrometer sensitivity using method A. The values of $q_{\mathrm{ref},t_{\min}}^{\#1}$, $q_{\mathrm{ref},t_{0.5}}^{\#1}$, and $q_{\mathrm{ref},t_{\max}}^{\#1}$ are the results for first‐circuit in RAMTEC Pro. The values of $q_{\mathrm{EUT},t_{\min}}^{\#1}$, $q_{\mathrm{EUT},t_{0.5}}^{\#1}$, and $q_{\mathrm{EUT},t_{\max}}^{\#1}$ are the results for first‐circuit in RAMTEC Duo and SuperMAX. The values of $q_{\mathrm{EUT},t_{\min}}^{\#2}$, $q_{\mathrm{EUT},t_{0.5}}^{\#2}$, and $q_{\mathrm{EUT},t_{\max}}^{\#2}$ are the results for second‐circuit in RAMTEC Pro, RAMTEC Duo, and SuperMAX. Coefficient *k*_elec,user_ is calculated based on *k*_elec_ for first‐circuit in RAMTEC Pro.

		RAMTEC Pro		RAMTEC Duo		SuperMAX	
Parameters		First‐circuit	Second‐circuit	First‐circuit	Second‐circuit	First‐circuit	Second‐circuit
*t*_ref,min_	[sec]	6	6	6	6	6	6
*t*_ref,0.5_	[sec]	64	64	64	64	64	64
*t*_ref,max_	[sec]	128	128	128	128	128	128
$q_{\mathrm{ref},t_{\min}}^{\#1}$	[nC]	0.94305	−	−	−	−	−
$q_{\mathrm{ref},t_{0.5}}^{\#1}$	[nC]	99.8572	−	−	−	−	−
$q_{\mathrm{ref},t_{\max}}^{\#1}$	[nC]	199.6918	−	−	−	−	−
$q_{\mathrm{EUT},t_{\min}}^{\#1}$	[nC]	−	−	0.9418	−	0.945	−
$q_{\mathrm{EUT},t_{0.5}}^{\#1}$	[nC]	−	−	99.689	−	100.0	−
$q_{\mathrm{EUT},t_{\max}}^{\#1}$	[nC]	−	−	199.486	−	199.9	−
$q_{\mathrm{EUT},t_{\min}}^{\#2}$	[nC]	−	0.94305	−	0.9418	−	0.947
$q_{\mathrm{EUT},t_{0.5}}^{\#2}$	[nC]	−	99.8036	−	99.740	−	99.94
$q_{\mathrm{EUT},t_{\max}}^{\#2}$	[nC]	−	199.6011	−	199.455	−	199.8
${\bar{r}}_{\mathrm{elec},t_{\min}}^{\#1}$		−	−	1.0013	−	0.9979	−
${\bar{r}}_{\mathrm{elec},t_{0.5}}^{\#1}$		−	−	1.0017	−	0.9986	−
${\bar{r}}_{\mathrm{elec},t_{max}}^{\#1}$		−	−	1.0010	−	0.9990	−
${\bar{r}}_{\mathrm{elec},t_{\min}}^{\#2}$		−	1.0000		1.0013	−	0.9958
${\bar{r}}_{\mathrm{elec},t_{0.5}}^{\#2}$		−	1.0005		1.0012	−	0.9992
${\bar{r}}_{\mathrm{elec},t_{max}}^{\#2}$		−	1.0005		1.0012	−	0.9995
${\bar{r}}_{\mathrm{elec}}^{\#1}$		−	−	1.0013	−	0.9985	−
${\bar{r}}_{\mathrm{elec}}^{\#2}$		−	1.0003	−	1.0012	–	0.9982
$k_{\mathrm{elec},\mathrm{user}}^{\#1}$		−	−	1.0014	−	0.9986	−
$k_{\mathrm{elec},\mathrm{user}}^{\#2}$		−	1.0004	−	1.0013	–	0.9983
*k*_elec_		1.0001	−	1.0006	−	0.9986	0.9988
*d*	[%]	−	0.030	0.080	0.070	0.0	−0.030

#1:First‐circuit.

#2:Second‐circuit.

The mean relative sensitivity, ${\bar{r}}_{\mathrm{elec}}^{\#1}$, calculated using Equation (4) was 1.0013 for RAMTEC Duo and 0.9985 for SuperMAX. For second‐circuit, ${\bar{r}}_{\mathrm{elec}}^{\#2}$ calculated using Equation (5) was 1.0003, 1.0012, and 0.9982 for RAMTEC Pro, RAMTEC Duo, and SuperMAX, respectively. Electrometer sensitivity coefficient $k_{\mathrm{elec},\mathrm{user}}^{\#1}$ calculated using Equation (6) was 1.0014 and 0.9986 for RAMTEC Duo and SuperMAX, respectively. For second‐circuit, $k_{\mathrm{elec},\mathrm{user}}^{\#2}$ calculated using Equation (7) was 1.0004, 1.0013, and 0.9983 for RAMTEC Pro, RAMTEC Duo, and SuperMAX, respectively. The maximum relative deviation (*d*) from *k*_elec_ was +0.080%.

Table 5 lists the parameters obtained using method B, in which the first‐circuit of every electrometer was used as the reference. Unlike method A, ${\bar{r}}_{\mathrm{elec}}^{\#2}$ was based on $q_{\mathrm{ref},t_{\min}}^{\#1}$, $q_{\mathrm{ref},t_{0.5}}^{\#1}$, and $q_{\mathrm{ref},t_{\max}}^{\#1}$ from the same device in method B. The calculated ${\bar{r}}_{\mathrm{elec}}^{\#2}$ values were 1.0003, 0.9999, and 0.9997 for RAMTEC Pro, RAMTEC Duo, and SuperMAX, respectively, and the $k_{\mathrm{elec},\mathrm{user}}^{\#2}$ values were 1.0004, 1.0005, and 0.9983, respectively. The maximum relative deviation from *k*_elec_ for method B was −0.050%.

**TABLE 5:** Parameters obtained from the intercomparison of electrometer sensitivity using method B. The values of $q_{\mathrm{EUT},t_{\min}}^{\#1}$, $q_{\mathrm{EUT},t_{0.5}}^{\#1}$, and $q_{\mathrm{EUT},t_{\max}}^{\#1}$ are the results for first‐circuit in RAMTEC Pro, RAMTEC Duo, and SuperMAX. The values of $q_{\mathrm{EUT},t_{\min}}^{\#2}$, $q_{\mathrm{EUT},t_{0.5}}^{\#2}$, and $q_{\mathrm{EUT},t_{\max}}^{\#2}$ are the results for second‐circuit in RAMTEC Pro, RAMTEC Duo, and SuperMAX. Coefficient *k*_elec,user_ of second‐circuit is calculated based on *k*_elec_ of first‐circuit for each of the three electrometers.

		RAMTEC Pro		RAMTEC Duo		SuperMAX	
Parameters		First‐circuit	Second‐circuit	First‐circuit	Second‐circuit	First‐circuit	Second‐circuit
*t*_ref,min_	[sec]	6	6	6	6	6	6
*t*_ref,0.5_	[sec]	64	64	64	64	64	64
*t*_ref,max_	[sec]	128	128	128	128	128	128
$q_{\mathrm{ref},t_{\min}}^{\#1}$	[nC]	0.94305	–	0.9418	–	0.945	–
$q_{\mathrm{ref},t_{0.5}}^{\#1}$	[nC]	99.8572	–	99.689	–	100.0	–
$q_{\mathrm{ref},t_{\max}}^{\#1}$	[nC]	199.6918	–	199.486	–	199.9	–
$q_{\mathrm{EUT},t_{\min}}^{\#1}$	[nC]	–	–	–	–	–	–
$q_{\mathrm{EUT},t_{0.5}}^{\#1}$	[nC]	–	–	–	–	–	–
$q_{\mathrm{EUT},t_{\max}}^{\#1}$	[nC]	–	–	–	–	–	–
$q_{\mathrm{EUT},t_{\min}}^{\#2}$	[nC]	–	0.94297	–	0.9418	–	0.947
$q_{\mathrm{EUT},t_{0.5}}^{\#2}$	[nC]	–	99.8464	–	99.740	–	99.94
$q_{\mathrm{EUT},t_{\max}}^{\#2}$	[nC]	–	199.6089	–	199.455	–	199.8
${\bar{r}}_{\mathrm{elec},t_{\min}}^{\#1}$		–	–	–	–	–	–
${\bar{r}}_{\mathrm{elec},t_{0.5}}^{\#1}$		–	–	–	–	–	–
${\bar{r}}_{\mathrm{elec},t_{max}}^{\#1}$		–	–	–	–	–	–
${\bar{r}}_{\mathrm{elec},t_{\min}}^{\#2}$		–	1.0001		1.0000		0.9979
${\bar{r}}_{\mathrm{elec},t_{0.5}}^{\#2}$		–	1.0001		0.9995		1.0006
${\bar{r}}_{\mathrm{elec},t_{max}}^{\#2}$		–	1.0004		1.0002		1.0005
${\bar{r}}_{\mathrm{elec}}^{\#1}$		–	–	1.0013	–	0.9985	–
${\bar{r}}_{\mathrm{elec}}^{\#2}$		–	1.0002	–	0.9999	–	0.9997
$k_{\mathrm{elec},\mathrm{user}}^{\#1}$		–	–	–	–	–	–
$k_{\mathrm{elec},\mathrm{user}}^{\#2}$		–	1.0003	–	1.0005	–	0.9983
*k*_elec_		1.0001	–	1.0006	–	0.9986	0.9988
*d*	[%]	–	0.020	–	−0.010	–	−0.030

#1:First‐circuit.

#2:Second‐circuit.

**FIGURE 6:**
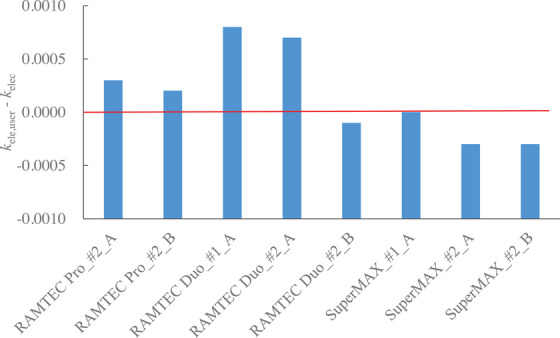
Relationship between the electrometers used in this study and the differences between *k*_elec_ and *k*_elec,user_. Electrometers are labeled according to the circuit they are based on and method A or B. For example, “RAMTEC Pro_#2_B” indicates the second‐circuit of RAMTEC Pro, method B (Figures 3 and 5).

Figure 6 shows the electrometers used in this study and the results of subtracting *k*_elec_ from *k*_elec,user_. The horizontal axis shows the electrometer, the circuit used for cross‐calibration, and the method used in this study. For example, “RAMTEC Pro_#2_B” indicates the second‐circuit of RAMTEC Pro, method B (Figures 3 and 5). For all electrometers, *d* calculated from Equation (8) was found to be within ± 0.1%. The maximum *d* was 0.0008 points. (Figure 6, RAMTEC Duo, first‐circuit, method A)

Table 6 lists the short‐term repeatability. It was confirmed that there were no dramatic changes over six‐month and that the results were stable, with only slight changes in the last significant digit.

**TABLE 6:** Evaluation of short‐term reproducibility. The short‐term stability of this study was evaluated by measuring every three months for six months.

	RAMTEC Pro		SuperMAX	
Date	${\bar{r}}_{\mathrm{elec}}^{\#2}$	$k_{\mathrm{elec},\mathrm{user}}^{\#2}$	${\bar{r}}_{\mathrm{elec}}^{\#2}$	$k_{\mathrm{elec},\mathrm{user}}^{\#2}$
February, 2025	1.0002	1.0003	0.9997	0.9983
May, 2025	1.0002	1.0003	0.9997	0.9983
August, 2025	1.0003	1.0004	0.9996	0.9982

Table 7 lists the budget sheet for this study. The expanded uncertainty (*k* = 2) was 0.40%. The largest uncertainty factor was the stability time, at 0.17%.

**TABLE 7:** Budget sheet for this study. Relative standard uncertainty was estimated using type A and type B features that affect the measurement results. The combined standard uncertainty was calculated as the square root of the sum of squares, and the expanded uncertainty was calculated using a coverage factor of *k* = 2.

Perfomance charactristic	Limit or standard uncertainty	Unit	Type	Relative standard uncertainty		Remarks/assumptions relating standard to conditions of measurement
Display resolution	0.005	nC	B	0.0029	%	The display resolution varies depending on the electrometer's specifications, but to avoid underestimating the uncertainty, half the maximum display resolution of 0.01 nC was used as the representative value.The sensitivity coefficient was calculated as the coefficient of variation when the sensitivity was changed by 0.01 nC.
Lnog‐term‐stability	0.1	%	B	0.058	%	1.5 years after calibration ^4^
Zero shift	0.1	%	B	0.058	%	Percentage of signal charge ^4^
Stability time	0.29	%	B	0.17	%	After 1 h response should be half way to final value ^4^
Temp. response	0.013	%	B	0.0075	%	Used within 5°C of calibration temperature ^4^
*q*_ref_	0.0042	%	A	0.0019	%	The worst‐case coefficient of variation was used as the limit. Measurements were repeated five times, and the estimate was made using type A.
*q*_EUT_	0.0047	%	A	0.0021	%	The worst‐case coefficient of variation was used as the limit. Measurements were repeated five times, and the estimate was made using type A.
*k*_elec_	0.08	%	B	0.080	%	The calibration certificate uncertainty (*k *= 1) was used as the representative value.
*u*_c_				0.20	%	
*U*(*k =* 2)				0.40	%	

∂u:Sensitivity coefficient.

*u*_c_:Combined standard uncertainty.

*U*:Expanded uncertainty.

## 4 DISCUSSION

Tsuno et al. reported that the coefficient of variation across repeated charge measurements using 12 linacs and a sample size of 1050 ranged between 0.01% and 0.07% over 10 trials, and the coefficient of variation for ionization chamber measurements under calibration conditions using a linac was below 0.1%.^9^ As listed in Table 3, the coefficient of variation obtained in this study was also below 0.1%, indicating that the measurements of electric charge were sufficiently precise with minimal variation.

Tables 4 and 5 clearly show that *k*_elec,user_ can be measured with an accuracy of less than 0.1% relative to *k*_elec_. Previous studies have suggested that *k*_elec_ may vary by as much as 0.25%,^8^ and the uncertainty of *k*_elec,user_ has been reported to be 0.18% (*k* = 1),^7^ and from Table 7, our relative standard uncertainty was 0.20 (*k* = 1), which was comparable to previous reports, supporting the high accuracy of our measurement results. Because the uncertainty in stability time was estimated to be the largest, we believe that further attention should be paid to future evaluations.

Furthermore, Tables 4 and 5 reveal a maximum difference of 0.0008 in *k*_elec,user_ (for second‐circuit in RAMTEC Duo) depending on the reference electrometer. This finding is supported by Figure 6, which shows agreement with the standard laboratory for all potential measurements within 0.1%. Considering an approximate uncertainty in *N*_D,w_ of 0.55%^8^ and that the beam quality conversion factor can have an uncertainty of approximately 1.5%,^10^, ^11^, ^12^ it is highly likely that the combined standard uncertainty for the absorbed dose‐to‐water calculated using the root‐sum‐of‐squares renders this difference negligible due to rounding. Although Table 5 shows a smaller relative deviation (−0.050%) and may yield slightly more accurate results due to the use of an identical circuit structure, the difference is minimal (only 0.0008 points), indicating that either *k*_elec_ or *k*_elec,user_ can be used without notably altering the absorbed dose measurement. Furthermore, the results in Table 6 show that our method is guaranteed to be stable over the short term.

When comparing the sensitivity of electrometers using two independent circuits, first‐circuit of either the same electrometer or a different electrometer can be used as the reference. Hence, second‐circuit can be calibrated locally through intercomparison at the facility, without depending on an external standards laboratory. In some cases, the calibration coefficient determined for first‐circuit may be applied to second‐circuit. However, in such cases, a thorough preliminary evaluation must be conducted to confirm that the performance of the electrometer has not been compromised,^4^ as previously discussed.^5^, ^6^, ^7^ The ability to use either method to compare the sensitivity of second‐circuit is a major achievement.

The uncertainty of absorbed dose‐to‐water measurements is attributed to the radiation conversion coefficient. Wang and Rogers used Monte Carlo simulations to demonstrate that the radiation quality conversion factor for ^6^ ^0^Co is 0.5% higher than the value recommended in the AAPM TG‐51 protocol and approximately 1% higher than that in the IAEA TRS‐398 code of practice.^13^ González–Castaño et al. compared beam conversion factors obtained from the EGSnrc software toolkit and experimental measurements. The uncertainty in the Monte Carlo simulations was 1%, whereas that in experimental measurements was 0.5%.^14^ As the uncertainty due to the radiation quality conversion coefficient can be gradually reduced over time, the contribution of the uncertainty due to the electrometer calibration constant *k*_elec_ will increase.

Although sensitivity comparisons between electrometers can be conducted using beams emitted from a linac,^5^ it requires two sets of ionization chambers and electrometers^5^, ^6^, ^7^ and cannot be performed during patient treatments.^7^ From a practical standpoint, our proposed method is advantageous in terms of operational efficiency. Given the extensive workload involved in quality assurance tasks,^15^ an efficient workflow is highly desirable. Our method offers a favorable balance between precision and operational efficiency, highlighting its value. Moreover, as our method allows in‐house calibration without relying on external laboratories, it may considerably contribute to reducing both economic and labor costs, providing a noteworthy contribution.

Because electrometers contain protection resistors, the current source establishes a series connection between its resistance and the protection resistor, potentially altering the total resistance. However, unless there are changes to the specifications, settings, or components of the electrometer, the protection resistor remains stable. Additionally, no impact due to the protection resistor was observed in the electrometers used in this study, indicating that this method is applicable to electrometers of the same model. For electrometers of other models, however, it remains unclear whether the protection resistor would affect the measurements. For example, caution is required in the following cases: This potential source has two built‐in resistors with resistance values that differ by 10 times. The protective resistance for each electrometer device is not disclosed and is not changed during normal use. However, if the protective resistance is changed when the electrometer is repaired, Ohm's law may cause the current flowing through the electrometer to change. If there is a change of 10 times or more compared to the previous current value, the protective resistance may have been changed, so it is recommended that you contact the manufacturer. Therefore, the accuracy of the sensitivity comparison results must be confirmed against calibration data from certified standards laboratories. Ensuring the validity of measurement results is necessary to maintain the accuracy of absorbed dose‐to‐water measurements beyond electrometer calibration.

## 5 CONCLUSION

We investigated the feasibility of determining the calibration coefficient of the second‐circuit in electrometers equipped with dual‐circuit systems through intercomparison of their responses. The sensitivity coefficients obtained by referencing first‐circuit of either the same or a different electrometer were comparable in accuracy to those provided by accredited standards laboratories. Thus, the second‐circuit of dual‐circuit electrometers can be calibrated in‐house through intercomparison without outsourcing to a calibration laboratory. Additionally, regarding the measurement uncertainty of the absorbed dose‐to‐water, the influence of the electrometer calibration coefficient *k*_elec_ is negligible. Therefore, for electrometers that are properly inspected and maintained in accordance with established standards, the calibration coefficient of first‐circuit may be applicable to second‐circuit. In this case, the following prerequisites must be met: the zero‐point drift and zero‐point shift must be smaller than the performance requirements, and the electrometer must be the one used in this research. Also, polarity dependence due to current in the first and second circuits must be acceptable. The presence or absence of polarity dependency can be confirmed by checking the calibration certificate of the electrometer used.

The custom‐designed current source used in this study does not rely on beams generated by a linac, thereby reducing the operational burden on the accelerator. This allows for quality control of electrometers to be conducted without disrupting patient treatments.

The electrometer intercomparison using our current source is accurate and operationally efficient. It provides a practical approach to calibration and quality assurance, offering an important advantage in terms of resource management and cost‐effectiveness.

## AUTHOR CONTRIBUTIONS

Hayato Tsuno devised the study and drafted the manuscript, supported by Koji Sasaki. Hayato Tsuno and Ruan Sasaki performed analysis and interpretation of data. Koji Sasaki, Kohei Nishi, Tae Ushikawa, Daisaku Goto, Yasuhiro Kawashima and Ririko Kosugi were involved in the study design and contributed significantly to the edition of the manuscript. All authors read and approved the final manuscript.

## CONFLICT OF INTEREST STATEMENT

There are no conflicts of interest to declare.

## Data Availability

The data that support the findings of this study are not available from the corresponding author, upon reasonable request.
